# Social and Poor Law Problems

**Published:** 1907-05-18

**Authors:** 


### SOCIAL AND POOR LAW PROBLEMS.

### SUGGESTED CHANGES IN THE POOR-LAW.

The Boards of Guardians throughout the country are considering the suggestions which they will lay before the Royal Commission on the Poor-laws when that body shall examine them. The proposals vary in different localities, but some poor-law problems are universal, and therefore we find that identical, or at least similar, suggestions come from several places. One very general proposal is that illegitimate children should be compelled to support their mothers. At present the illegitimate child is free from the liabilities of the child born in wedlock, which may indeed be but a poor compensation for the stigma under which he lives, but is certainly unjust to the more respectable poor. This change, if carried, may, however, affect other than paupers. At present the mother of an illegitimate cannot benefit under the Workmen's Compensation Act if by the death of her child she is deprived of her means of support. If the liability of illegitimate children in law is accepted in the case of parish relief, it would probably have to be extended to any case where the child had been voluntarily supporting his mother and she was deprived of his support through such an accident as would justify her, if a married woman, claiming compensation under the Act. The question of illegitimacy has a bearing also on the proposals made by several Boards to seek compulsory powers to detain in the workhouse women of loose character—a phrase which may be taken to mean mothers of more than one illegitimate child. It is proposed to keep these in the workhouse for periods varying from one to three years. In the case of women of absolutely immoral nature this might only have the effect of preventing their coming into the workhouse for their confinements. Of more definite value is the proposal to seek powers to detain feeble-minded men and women. The mischief done to the race by feeble-minded persons becoming parents can hardly be reckoned, and it would be better for themselves, as well as for the nation, that when they come under the Poor-law they should be protected against their own weaknesses. Another point from which most parishes suffer is the improvidence of pensioners—army and others—who receive their pensions quarterly, spend all in a short time, and come upon the Guardians for support until the next instalment is due. The Carmarthen Guardians would like to have pensions paid monthly, while those of Gloucester went farther and suggested weekly payment, but this latter proposal was finally given up as impracticable. Other suggestions deal with the provision for epileptics, but are of various kinds. Aberystwith desires that separate homes should be provided for groups of small workhouses for the reception of epileptics, Carmarthen specifies only that epileptics should be kept at some place other than the workhouse; Gloucester seeks compulsory power to send them to isolation homes; while Chesterfield would like a Government grant of 4s. per week towards the maintenance of imbeciles and epileptics detained in workhouses. The Bourne Guardians desire the power to order people to the workhouse, and those of Carmarthen would like at least to have the power to remove to the workhouse sick paupers who could not be properly attended to outside. Of course it is not likely that all the suggestions made will be accepted by the Commission; but the knowledge which its members will gain from the opinions of those who have to deal with the difficult problems of the poor and their needs will doubtless have a useful effect.

